# Strategies for chronic coronary disease: A brief guide for clinicians

**DOI:** 10.1038/s44325-024-00006-w

**Published:** 2024-06-04

**Authors:** Chayakrit Krittanawong, Muzamil Khawaja, Hafeez Ul Hassan Virk, Johao Escobar, Umair Khalid, Yochai Birnbaum, Carl J. Lavie, Samin Sharma, Hani Jneid, Sunil Rao, Salim S. Virani

**Affiliations:** 1 Cardiology Division, NYU Langone Health and NYU School of Medicine, New York, NY USA; 2 Cardiology Division, Emory University School of Medicine, Atlanta, GA USA; 3 Harrington Heart & Vascular Institute, Case Western Reserve University, University Hospitals Cleveland Medical Center, Cleveland, OH USA; 4 Division of Cardiology, Harlem Cardiology, New York, NY USA; 5 Michael E. DeBakey VA Medical Center, Section of Cardiology, Baylor College of Medicine, Houston, TX USA; 6 Section of Cardiology, Department of Medicine, Baylor College of Medicine, Houston, TX USA; 7 John Ochsner Heart and Vascular Institute, Ochsner Clinical School, The University of Queensland School of Medicine, New Orleans, LA USA; 8 Cardiac Catheterization Laboratory of the Cardiovascular Institute, Mount Sinai Hospital, New York, NY USA; 9 Division of Cardiology, University of Texas Medical Branch, Houston, TX 77002 USA; 10 Office of the Vice Provost, The Aga Khan University, Karachi, 74800 Pakistan

**Keywords:** Cardiology, Interventional cardiology

## Abstract

The 2023 Multisociety Guidelines for the Management of Patients with Chronic Coronary Disease (CCD) is a collaborative effort between the American Heart Association (AHA) and the American College of Cardiology (ACC) that provides recommendations on the management of this condition. Efficient management of CCD involves non-pharmaceutical interventions that promote healthier lifestyles, such as increasing physical activity, adopting a balanced diet, and addressing tobacco misuse. These changes are critical to improving cardiovascular outcomes for individuals with CCD. In addition to lifestyle modifications, pharmacological and revascularization treatments also play an essential role in managing CCD. These treatments target the complex mechanisms of the disease, optimize cardiac function, and decrease the risk of adverse events. The combination of lifestyle changes and medicine-based medications enhances the quality of life and lowers mortality rates among individuals with CCD. This article review emphasizes the importance of non-pharmacological and pharmacological strategies that align with the AHA/ACC guidelines. In addition, the primary objective of this study is to enhance comprehension of the approaches that have led to better cardiovascular results for patients diagnosed with CCD.

## Introduction

The 2023 Multisociety Guideline for the Management of Patients with Chronic Coronary Disease is a collaborative effort between the American Heart Association (AHA) and the American College of Cardiology (ACC). This guideline represents an essential tool for medical professionals that provides the best medicine-based recommendation in the management of patients with chronic coronary disease (CCD). In response to a terminology modification, the guideline adopts “chronic coronary disease” instead of “stable ischemic heart disease,” consistent with the European Society of Cardiology’s (ESC) update^1^.

The 2023 AHA/ACC Guidelines apply the term CCD in the outpatient setting for individuals with left ventricular (LV) systolic dysfunction due to coronary artery disease (CAD) or acute coronary syndrome (ACS) with or without revascularization once all acute cardiovascular concerns have been resolved. Furthermore, CCD includes patients diagnosed with coronary disease solely through noninvasive tests and based on the clinician’s judgment. Additionally, CCD involves persistent angina syndromes linked to various underlying causes, including ischemia, coronary vasospasm, or microvascular angina^2^. CCD has profound implications for the healthcare system around the globe. Approximately 20.1 million Americans have CCD, highlighting the seriousness of this health problem. Additionally, data has revealed that a quarter of all U.S. myocardial infarctions (MI) occur within the subset of CCD patients with a previous history of MI. Therefore, the urgency for practical management approaches is of the utmost interest. Despite a promising 25% reduction in coronary heart disease (CHD) mortality over the past decade, CHD remains the leading cause of national and global mortality. This problem’s magnitude extends beyond personal spheres, encompassing economic and societal burdens^2^. Thus, a comprehensive interpretation of these outcomes is essential to understand the significant challenges commonly seen among patients with CCD.

This article review emphasizes the importance of nonpharmacological and pharmacological methods, aligning with the AHA/ACC guidelines. This holistic approach aims to enhance comprehension of the diverse strategies that collectively improve cardiovascular outcomes for individuals affected by CCD.

## Nonpharmacological therapies in patients with CCD

Lifestyle modification is a crucial component in managing cardiovascular disease. The impact of sedentary habits on CCD risk cannot be overstated, magnifying physical activity’s pivotal role in promoting cardiac health and prolonging life. Furthermore, the addition of healthy diets and tobacco abuse reduction can improve cardiovascular outcomes among this population.

### Physical activity and diet

Physical inactivity is a significant risk factor for CCD^3,4^. Physical activity prolongs life expectancy and delays cardiovascular disease development by almost 3 years^5^. In patients with CCD, physical activity lowers the risk of major cardiovascular events (MACE), hospitalization, and costs related to medical care and enhances quality of life^6^. More critically, physical activity in individuals with CCD improves oxygen consumption, especially with high-intensity training sessions in the first eight weeks^7^. The decision to prescribe physical activity in CCD individuals is challenging. Among various methods, the primary aspect of utmost importance is the overall caloric expenditure rather than the specific physical modality. A caloric expenditure greater than 1500 Kcal per day has been shown to delay CCD progression^8,9^. Therefore, when devising physical activity plans for cardiac rehabilitation, it is essential to prioritize the exercise modality that has greater adherence among patients with this condition.

Diets, such as the Mediterranean diet (MD), Dietary Approaches to Stop Hypertension (DASH) diet, and others, can positively impact patients with CCD. The MD consists of plant-based foods. It emphasizes consuming minimally processed foods, dairy products, and a higher intake of olive oil, fish, and poultry in combination with moderate wine ingestion^10^. Notably, MD can help modulate endothelial function in patients with CCD^11^. It has also been shown that MD decreases the risk of CCD by 30%^12^. On the other hand, the DASH diet has been associated with a substantial CCD reduction^13^. The DASH diet highlights the importance of a balanced diet consisting of fresh vegetables, fat-free/low-fat dairy products, whole grains, nuts, and legumes. This diet regimen sets restrictions on the intake of total and saturated fats, cholesterol, red and processed meats, sweets, added sugars, and sugar-sweetened drinks^14^. The DASH diet decreases high-sensitivity C-reactive protein (HS-CRP) and CXCL4 levels, a chemokine linked to thrombocyte activation, and has a protective effect against atherosclerosis^15–19^. It should be noticed that a meta-analysis demonstrated that vegetarian diets are related to a lower incidence of CCD^20^.

Studies have established a correlation between the consumption of nuts and extra-virgin olive oil and the inhibition of multiple mechanisms, including inflammatory pathways that are responsible for atherosclerosis^21,22^. However, this strategy has not successfully reduced inflammatory markers in patients with CCD^23^. Interestingly, in a randomized controlled trial (RCT) among CCD patients, the addition of nuts to a healthy diet decreased non-high-density lipoprotein cholesterol (HDL-C) levels, but no effects on low-density lipoprotein cholesterol (LDL-C) were reported^24^.

Some diets have been demonstrated to obtain better outcomes in CCD patients. As an example, the CORDIOPREV study compared the effects of the MD and a low-fat diet in patients with CCD. The trial found that the MD was superior in preventing MACE, such as MI, revascularization, stroke, lower extremity peripheral artery disease, and cardiovascular death, especially in men^25^. In the EVADE CAD trial, the HS-CRP reduction was 32% lower with a vegan diet than the AHA diet^26^. Even though no RCTs have been issued about the effects of the ketogenic diet on CCD, this diet has been associated with a higher risk of MACE among this population^27^.

According to the 2023 AHA/ACC Guidelines, it is highly recommended that patients with CCD develop healthy eating patterns and maintain a consistent physical activity routine. Individuals with this condition should consider integrating regular physical activity, including aerobic and resistance exercises, while minimizing prolonged periods of sitting. For those eligible, engagement in cardiac rehabilitation provides significant cardiovascular benefits, leading to notable decreases in complications and mortality rates^2^.

In summation, the significance of adopting a healthy lifestyle, encompassing both diet and physical activity, cannot be overstated in the management and even prevention of CCD. Since physical inactivity poses such a substantial risk for CCD, it is essential to emphasize the pivotal role of regular exercise in mitigating this cardiovascular condition. Incorporating aerobic and resistance exercises, alongside minimizing prolonged periods of sitting, is crucial for individuals with CCD. Dietary choices play an equally critical role, with evidence supporting the positive impact of specific diets like the MD and DASH diets in reducing the risk of CCD and preventing MACEs. These diets, rich in plant-based foods and emphasizing balanced nutrition, contribute to improved endothelial function and decreased inflammation. The adoption of healthy eating patterns and an active lifestyle, as recommended by the 2023 AHA/ACC Guidelines, not only aids in managing CCD but also serves as a potent preventive measure, promoting overall cardiovascular health and enhancing the quality of life.

### Tobacco and electronic cigarettes

The ACC/AHA Guidelines state that CCD patients must quit smoking^2^. It is known that certain groups (e.g. younger individuals with limited income) may experience psychiatric conditions like anxiety, depression, and anger at increased rates. In addition, these patients may lack a support system, which increases their susceptibility to engaging in smoking behavior^28^. It is worth mentioning that the risk of CCD remains present for two decades after tobacco abuse cessation^29^. After counseling, individuals who want to quit smoking can choose from three primary pharmacological options: nicotine replacement therapy (NRT), varenicline, and bupropion^30^. Notably, varenicline and bupropion have not been associated with an increased risk or worsening of neuropsychiatric conditions compared to nicotine patches or placebo^31,32^.

Even though electronic cigarettes have shown an improved likelihood of facilitating successful smoking cessation in comparison with NRT, the ACC/AHA warrants caution in using them as the primary method for quitting smoking, given the current lack of comprehensive, long-term safety data and the potential risks of prolonged use^2^.

To summarize, the use of tobacco and electronic cigarettes poses a grave concern for patients with CCD. Smoking tobacco is a well-established risk factor for the development and progression of CCD, significantly increasing the likelihood of adverse cardiovascular events. The harmful effects of tobacco smoke, including the presence of various toxic chemicals, contribute to inflammation, endothelial dysfunction, and accelerated atherosclerosis, further exacerbating the cardiovascular burden in individuals with CCD. Additionally, while electronic cigarettes are often marketed as a less harmful alternative to traditional tobacco, emerging evidence suggests potential adverse effects on cardiovascular health. The inhalation of electronic cigarette aerosols may still pose risks to individuals with CCD, as the aerosols contain harmful substances that can impact vascular function. As such, it is strongly advised for patients with CCD to avoid both tobacco and electronic cigarette use.

## Pharmacological intervention in patients with CCD

**Fig. 1:**
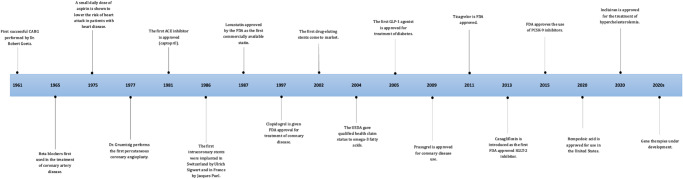
A timeline of treatment strategies for chronic coronary disease. A timeline of treatment strategies using pharmacological approach and revascularization approach for chronic coronary disease from 1961 to 2024.

**Fig. 2:**
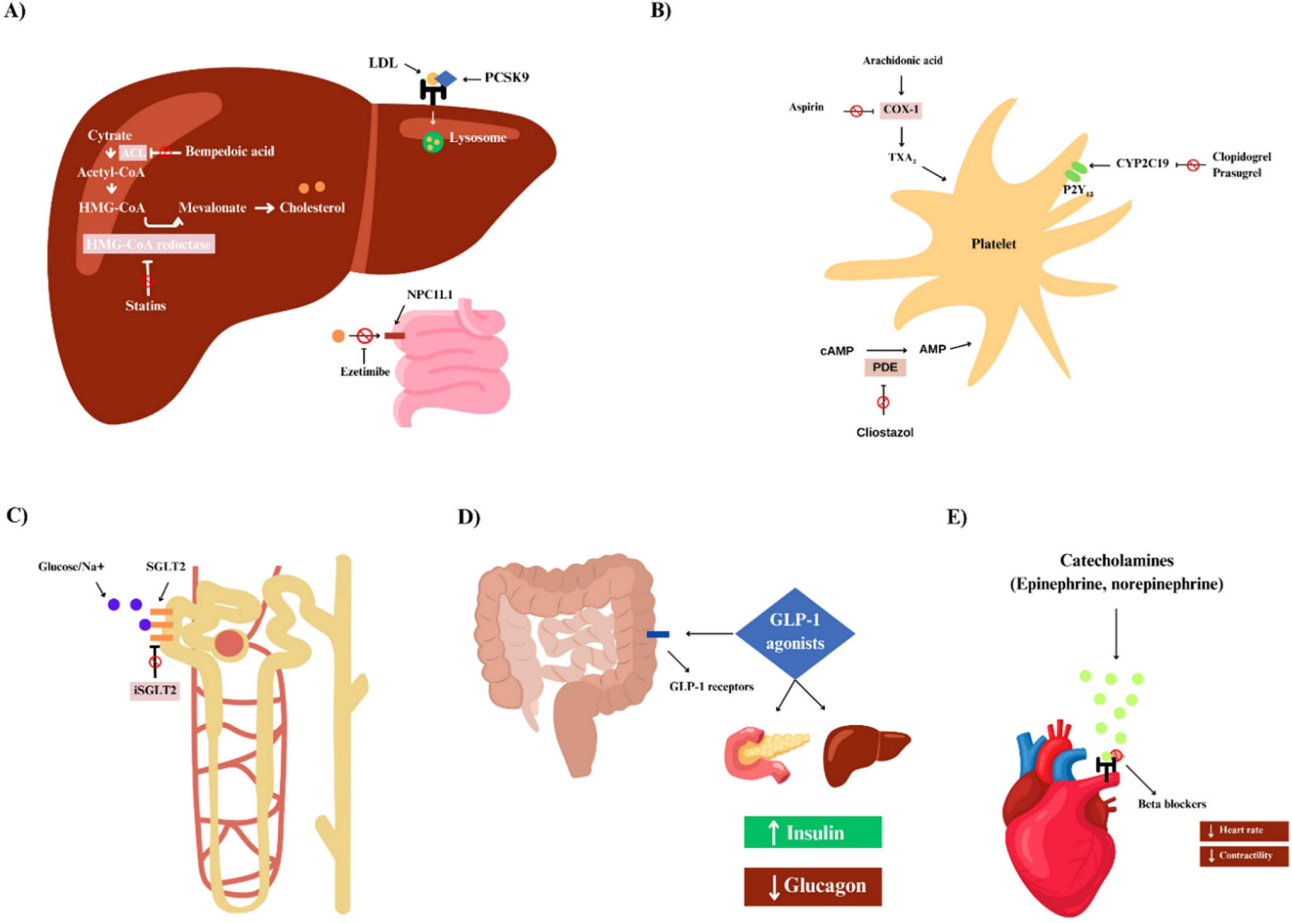
Action mechanisms of pharmacological therapies in chronic coronary disease. **A** Bempedoic acid reduces cholesterol synthesis by inhibiting ATP citrate lyase, an enzyme involved in the production of cholesterol in the liver. Stains act by inhibiting an enzyme in the liver called HMG-CoA reductase. Ezetimibe inhibits intestinal cholesterol absorption by targeting the NPC1L1 protein. **B** Aspirin acts by inhibiting enzymes, mainly COX-1 and COX-2, responsible for producing prostaglandins. P2Y12 inhibitors block platelet activation by binding to P2Y12 receptors on platelet surfaces, inhibiting ADP-mediated signaling. **C** SGLT-2 inhibitors block sodium-glucose co-transporter 2 in renal tubules, reducing glucose reabsorption and increasing urinary glucose excretion. **D** GLP-1 agonists function by activating the GLP-1 receptor, enhancing glucose-dependent insulin secretion, suppressing glucagon release, slowing gastric emptying, and promoting satiety. **E** Beta-blockers function by blocking the activation of beta and alpha-adrenergic receptors in the heart and vasculature, leading to reduced heart rate, lowered blood pressure, and decreased myocardial oxygen demand.

In CCD management, several pharmacological options are fundamental to improving individual outcomes (Fig. 1). These strategies involve using specific drugs to target the complex physiological mechanisms of CCD. In addition, some treatments have inherent properties that help with other problematic consequences of CCD. The goal is to lessen the burden of ischemia, enhance cardiac function, and lower the risk of adverse cardiac-related events, meanwhile improving quality of life and reducing mortality (Fig. 2). A summary of all the pivotal trials for pharmacological trials is provided in Table 1.

**Table 1:** A summary of all the pivotal trials for pharmacological trials

Trial	*N* (Number of patients)	Methodology	Follow Up	Results
TNT	10,001	Patients with CAD and LDL-C < 130 mg/dL were randomly assigned to double-blind therapy and received either 10 mg or 80 mg of atorvastatin daily	4.9 years	Atorvastatin 80 mg resulted in significantly lower LDL-C levels and an absolute and relative reduction in the rate of MACEs
FOURIER	27,564	Patients with atherosclerotic cardiovascular disease and LDL cholesterol levels ≥ 70 mg/dL were randomly assigned to receive evolocumab inhibitor or placebo.	48 weeks	Evolocumab therapy, relative to placebo, significantly reduced the composite endpoint of cardiovascular death, myocardial infarction, stroke, hospitalization for unstable angina, or coronary revascularization.
PACMAN-AMI	300	Patients were randomized to receive alirocumab or placebo, initiated less than 24 hours after urgent PCI of a culprit lesion for MI, in addition to high-intensity statin therapy	52 weeks	The addition of alirocumab, compared with placebo, to high-intensity statin therapy resulted in significantly greater coronary plaque regression in non–infarct-related arteries after 52 weeks.
ODYSSEY OUTCOMES	18,925	Patients who had an acute coronary syndrome 1 to 12 months earlier, had a LDL-C level of ≥ 70 mg/dL, a non−HDL-C ≥ 100 mg/dL, or an apolipoprotein B level of ≥ 80 mg/dL, and were receiving statin therapy at a high-intensity dose or at the maximum tolerated dose randomly assigned to receive alirocumab or matching placebo	2.8 years	Among patients who had a previous acute coronary syndrome and who were receiving high-intensity statin therapy, the risk of recurrent ischemic cardiovascular events was lower among those who received alirocumab than among those who received placebo.
ORION-4*	~15,000	Patients with pre-existing atherosclerotic cardiovascular disease will be randomized to either inclisiran or matching placebo	~5 years	Results pending
CLEAR	13,970	Patients who were “statin-intolerant” and at high risk for, cardiovascular disease were assigned to receive oral bempedoic acid or placebo.	40.6 months	Treatment with bempedoic acid was associated with a lower risk of major adverse cardiovascular events (death from cardiovascular causes, nonfatal myocardial infarction, nonfatal stroke, or coronary revascularization).
STRENGTH	13,078	Adult patients (≥18 years) considered at high risk for a future cardiovascular event were randomized to receive 4 g/d of omega-3 fatty acids or corn oil	42 months	Among statin-treated patients at high cardiovascular risk, the addition of omega-3 CA, compared with corn oil, to usual background therapies resulted in no significant difference in a composite outcome of major adverse cardiovascular events.
REDUCE-IT	8179	Patients with established cardiovascular disease or with diabetes and other risk factors, who had been receiving statin therapy and who had a fasting triglyceride level of 135 to 499 mg/dL and a LDL-C level of 41 to 100 mg/dL were randomly assigned to receive icosapent ethyl or placebo	4.9 years	Among patients with elevated triglyceride levels despite the use of statins, the risk of ischemic events, including cardiovascular death, was significantly lower among those who received icosapent ethyl than among those who received placebo.
EVAPORATE	80	Patients with coronary atherosclerosis as documented by CCTA (one or more angiographic stenoses with ≥20% narrowing), on statin therapy, and with persistently elevated triglyceride levels were randomized to icosapent ethyl or placebo groups	18 months	Icosapent ethyl demonstrated significant regression of low attenuation plaque volume on CCTA compared with placebo over 18 months.
JELIS	18,645	Patients with total cholesterol of 6·5 mmol/L or greater were randomly assigned to receive either eicosapentaenoic daily with statin or statin only.	4.6 years	In patients with no history of CAD, eicosapentaenoic acid treatment reduced major coronary events by 18%, but this finding was not significant in the Eeicosapentaenoic acid group vs in the control group
CHARISMA	15,603	Patients with either clinically evident cardiovascular disease or multiple risk factors were randomized to receive clopidogrel (75 mg per day) plus low-dose aspirin (75 to 162 mg per day) or placebo plus low-dose aspirin	28 months	There was a suggestion of benefit with clopidogrel treatment in patients with symptomatic atherothrombosis and a suggestion of harm in patients with multiple risk factors. Overall, clopidogrel plus aspirin was not significantly more effective than aspirin alone in reducing the rate of myocardial infarction, stroke, or death from cardiovascular causes
PEGASUS	21,162	Patients who had had a myocardial infarction 1 to 3 years earlier were randomized to low-dose aspirin + ticagrelor at a dose of 90 mg twice daily, ticagrelor at a dose of 60 mg twice daily, or placebo.	33 months	In patients with a myocardial infarction more than 1 year previously, treatment with ticagrelor significantly reduced the risk of cardiovascular death, myocardial infarction, or stroke and increased the risk of major bleeding.
LEADER	9340	Patients with type 2 diabetes and high cardiovascular risk were randomized to receive liraglutide or placebo.	3.8 years	The rate of the first occurrence of death from cardiovascular causes, nonfatal myocardial infarction, or nonfatal stroke among patients with type 2 diabetes mellitus was lower with liraglutide than with placebo.
SUSTAIN-6	3297	Patients with type 2 diabetes who were on a standard-care regimen were randomized to receive semaglutide or placebo	109 weeks	In patients with type 2 diabetes who were at high cardiovascular risk, the rate of cardiovascular death, nonfatal myocardial infarction, or nonfatal stroke was significantly lower among patients receiving semaglutide than among those receiving placebo
REWIND	9901	Men and women aged at least 50 years with type 2 diabetes who had either a previous cardiovascular event or cardiovascular risk factors were randomly assigned to either dulaglutide or placebo.	5.4 years	Dulaglutide use resulted in significantly less first occurrence of the composite endpoint of non-fatal myocardial infarction, non-fatal stroke, or death from cardiovascular causes
EXSCEL	14,752	Patients with type 2 diabetes, with or without previous cardiovascular disease, were randomized receive exenatide or a matching placebo	3.2 years	Among patients with type 2 diabetes with or without previous cardiovascular disease, the incidence of major adverse cardiovascular events did not differ significantly between patients who received exenatide and those who received placebo.
ELIXA	6068	Patients with type 2 diabetes who had had a myocardial infarction or who had been hospitalized for unstable angina within the previous 180 days were randomized to receive lixisenatide or placebo in addition to locally determined standards of care.	25 months	In patients with type 2 diabetes and a recent acute coronary syndrome, the addition of lixisenatide to usual care did not significantly alter the rate of major cardiovascular events or other serious adverse events.
EMPA-REG OUTCOME	7020	Patients with diabetes and established cardiovascular disease were randomized to receive 10 mg or 25 mg of empagliflozin or placebo once daily	3.1 years	Patients with type 2 diabetes at high risk for cardiovascular events who received empagliflozin, as compared with placebo, had a lower rate of the primary composite cardiovascular outcome and of death from any cause when the study drug was added to standard care.
CANVAS PROGRAM	10,142	Patients with type 2 diabetes and high cardiovascular risk were randomly assigned to receive canagliflozin or placebo	188.2 weeks	Patients treated with canagliflozin had a lower risk of cardiovascular events than those who received placebo
DECLARE-TIMI 58	17,160	Patients with type 2 diabetes who had or were at risk for atherosclerotic cardiovascular were randomized to receive dapagliflozin or placebo.	4.2 years	Treatment with dapagliflozin did not result in a higher or lower rate of MACE than placebo but did result in a lower rate of cardiovascular death or hospitalization for heart failure
CREDENCE	4401	Patients with type 2 diabetes and albuminuric chronic kidney disease were randomized to receive canagliflozin at a dose of 100 mg daily or placebo.	2.62 years	The risk of kidney failure and cardiovascular events was lower in the canagliflozin group than in the placebo group
VERTIS CV	8246	Patients with type 2 diabetes and atherosclerotic cardiovascular disease were randomized to receive 5 mg or 15 mg of ertugliflozin or placebo once daily.	3.5 years	Ertugliflozin was non-inferior to placebo with respect to major adverse cardiovascular events.
HOPE	651	High-risk patients (55 years of age or older) who had evidence of vascular disease or diabetes plus one other cardiovascular risk factor and who were not known to have a low ejection fraction or heart failure were randomly assigned to receive ramipril (10 mg once per day orally) or matching placebo	5 years	Ramipril significantly reduces the rates of death, myocardial infarction, and stroke in a broad range of high-risk patients who are not known to have a low ejection fraction or heart failure.
EUROPA	12,218	Patients with stable CAD and no history of heart failure were randomized to perindopril 8 mg once daily or matching placebo	4.2 years	Perindopril significantly reduced the primary endpoint of cardiovascular death, myocardial infarction, or cardiac arrest.
QUIET	1750	Patients with stable CAD and no history of heart failure were randomized to quinapril 20 mg/day or placebo	27 months	Quinapril 20 mg did not significantly affect the overall frequency of clinical outcomes or the progression of coronary atherosclerosis.
CAMELOT	1991	Patients with angiographically documented CAD (>20% stenosis by coronary angiography) and diastolic blood pressure <100 mm Hg were randomized to receive amlodipine, 10 mg; enalapril, 20 mg; or placebo.	24 months	Administration of amlodipine to patients with CAD and normal blood pressure resulted in reduced adverse cardiovascular events. Directionally similar, but smaller and nonsignificant, treatment effects were observed with enalapril.
ISCHEMIA	5179	Patients with moderate or severe ischemia were randomized to an initial invasive strategy (angiography and revascularization when feasible) and medical therapy or to an initial conservative strategy of medical therapy alone and angiography if medical therapy failed	3.2 years	No evidence that an initial invasive strategy, as compared with an initial conservative strategy, reduced the risk of ischemic cardiovascular events or death from any cause
EXCEL	1905	Patients with left main CAD of low or intermediate anatomical complexity were randomized to undergo either PCI or CABG	5 years	No significant difference between PCI and CABG with respect to the rate of the composite outcome of death, stroke, or myocardial infarction
COURAGE	2287	Patients with stable CAD were randomly assigned to we assigned to undergo PCI with optimal medical therapy or optimal medical therapy alone	4.6 years	PCI did not reduce the risk of death, myocardial infarction, or other major cardiovascular events when added to optimal medical therapy.
BARI2D	2368	Patients with both type 2 diabetes and heart disease were randomized to undergo either prompt revascularization with intensive medical therapy or intensive medical therapy alone and to undergo either insulin-sensitization or insulin-provision therapy.	5 years	There was no significant difference in the rates of death and major cardiovascular events between patients undergoing prompt revascularization and those undergoing medical therapy or between strategies of insulin sensitization and insulin provision
REVIVED-BCIS2	700	Patients with a left ventricular ejection fraction of 35% or less, extensive CAD amenable to PCI, and demonstrable myocardial viability were randomized to a strategy of either PCI plus optimal medical therapy or optimal medical therapy alone	41 months	Revascularization by PCI did not result in a lower incidence of death from any cause or hospitalization for heart failure.
CASS	780	Patients with stable ischemic heart disease were randomly assigned to receive surgical or nonsurgical treatment for CAD	10 years	No significant difference in survival rates between patients treated with medical therapy alone and those treated with CABG over (aside from certain cases such as multivessel CAD and left main CAD)
STICH	1212	Patients with an ejection fraction of 35% or less and coronary artery disease amenable to CABG were randomly assigned to medical therapy alone or medical therapy plus CABG	56 months	There was no significant difference between medical therapy alone and medical therapy plus CABG with respect to the primary end point of death from any cause. Patients assigned to CABG, as compared with those assigned to medical therapy alone, had lower rates of death from cardiovascular causes and of death from any cause or hospitalization for cardiovascular causes.

### Lipid-lowering therapies

The 2023 AHA/ACC guidelines state that statin therapy is the primary strategy for lowering lipid levels in individuals with CCD. The guidelines consider additional options, such as ezetimibe, proprotein convertase subtilisin/kexin type 9 (PCSK-9) inhibitors, and bempedoic acid for specific patient populations (Table 2). Nevertheless, there is a need for more clinical data for newer medications like inclisiran^2^.

**Table 2:** Drugs Used in Patients with Chronic Coronary Disease: ESC and AHA/ACC Guidelines

Therapy	ESC Guideline	AHA/ACC Guideline
**Lipid Management: Statins**	Statins are recommended in all patients with CCS.	In patients with CCD, high-intensity statin therapy is recommended with the aim of achieving a ≥ 50% reduction in LDL-C levels to reduce the risk of MACE.
	If a patient’s goal is not achieved with the maximum tolerated dose of statin, a combination with ezetimibe is recommended.	In patients in whom high-intensity statin therapy is contraindicated or not tolerated, moderate-intensity statin therapy is recommended with the aim of achieving a 30% to 49% reduction in LDL-C levels to reduce the risk of MACE.
	For patients at very high risk who do not achieve their goal on a maximum tolerated dose of statin and ezetimibe, a combination with a PCSK9 inhibitor is recommended.	In patients with CCD who are judged to be at very high risk and who have an LDL-C level ≥70 mg/dL (≥1.8 mmol/L), or a non–high-density lipoprotein cholesterol (HDL-C) level ≥100 mg/dL (≥2.6 mmol/L), on maximally tolerated statin and ezetimibe, a PCSK9 monoclonal antibody can be beneficial to further reduce the risk of MACE.
		In patients with CCD on maximally tolerated statin therapy with an LDL-C level <100 mg/dL (<2.6 mmol/L) and a persistent fasting tri-glyceride level of 150 to 499 mg/dL (1.7–5.6 mmol/L) after addressing secondary causes, icosapent ethyl may be considered to further reduce the risk of MACE and cardiovascular death.
**Blood Pressure Management**	It is recommended that office BP is controlled to target values: systolic BP 120–130 mmHg in general and systolic BP 130–140 mmHg in older patients (aged >65 years).	In adults with CCD, nonpharmacologic strategies are recommended as first-line therapy to lower BP in those with elevated BP (120–129/ < 80 mm Hg).
	In hypertensive patients with a recent MI, betablockers, and RAS blockers are recommended.	In adults with CCD who have hypertension, a BP target of <130/<80 mm Hg is recommended to reduce CVD events and all-cause death.
	In patients with symptomatic angina, beta-blockers and/or CCBs are recommended.	In adults with CCD and hypertension (systolic BP ≥ 130 and/or diastolic BP ≥ 80 mm Hg), in addition to nonpharmacological strategies, GDMT angiotensin-converting enzyme (ACE) inhibitors, angiotensin-receptor blockers (ARB), or beta blockers are recommended as first-line therapy for compelling indications (eg, recent MI or angina), with additional antihypertensive medications (eg, dihydropyridine calcium channel blockers [CCB], long-acting thiazide diuretics, and/or mineralocorticoid receptor antagonists) added as needed to optimize BP control.
**SGLT2 inhibitors and GLP-1 agonists**	Treatment with ACE inhibitor is recommended in CCD patients with diabetes for event prevention.	In patients with CCD and heart failure with LVEF ≤ 40%, the use of an SGLT2 inhibitor is recommended to reduce the risk of cardiovascular death and heart failure hospitalization and to improve QOL, irrespective of diabetes status.
	The sodium-glucose co-transporter 2 inhibitors empagliflozin, canagliflozin, or dapagliflozin are recommended in patients with diabetes and CVD.	In patients with CCD and heart failure with LVEF > 40%, the use of an SGLT2 inhibitor can be beneficial in decreasing heart failure hospitalizations and improving QOL, irrespective of diabetes status.
	A glucagon-like peptide-1 receptor agonist (liraglutide or semaglutide) is recommended in patients with diabetes and CVD.	
**Beta blockers**	The use of beta-blocker therapy is recommended in patients with LV dysfunction or systolic HF.	In patients with CCD and LVEF ≤ 40% with or without previous MI, the use of beta-blocker therapy is recommended to reduce the risk of future MACE, including cardiovascular death.
	The usage of beta-blockers can help relieve angina and reduce morbidity and mortality in heart failure.	In patients with CCD and LVEF < 50%, the use of sustained-release metoprolol succinate, carvedilol, or bisoprolol with titration to target doses is recommended in preference to other beta blockers.
	Long-term therapy with oral beta-blockers should be taken into consideration in patients with previous STEMI.	In patients with CCD who were initiated on beta-blocker therapy for previous MI without a history of or current LVEF ≤ 50%, angina, arrhythmias, or uncontrolled hypertension, it may be reasonable to reassess the indication for long-term (>1 year) use of beta-blocker therapy for reducing MACE.
		n patients with CCD without previous MI or LVEF ≤ 50%, the use of beta-blocker therapy is not beneficial in reducing MACE, in the absence of another primary indication for beta-blocker therapy
**RAAS inhibitors**	ACE inhibitors (or ARBs) are recommended if a patient has other conditions (heart failure, hypertension, or diabetes) to reduce cardiovascular events.	In patients with CCD who also have hypertension, diabetes, LVEF ≤ 40% or CKD, the use of ACE inhibitors, or ARBs, is recommended to reduce cardiovascular events.
**Antiplatelet therapy**	Aspirin 75–100 mg daily is recommended in patients with a previous MI or revascularization Clopidogrel 75 mg daily is a recommended alternative to aspirin in those with aspirin intolerance DAPT (aspirin + clopidogrel) is recommended for 6 months following PCI in CCD (unless a shorter duration is indicated due to risk or the occurrence of life-threatening bleeding.	In patients with no indication for oral anticoagulant therapy, low-dose aspirin 81 mg (75–100 mg) is recommended to reduce atherosclerotic events Patients treated with PCI, DAPT (aspirin + clopidogrel) for 6 months followed by SAPT is indicated to reduce MACE and bleeding events. In patients treated with PCI who require anticoagulant therapy, DAPT for 1–4 weeks followed by clopigorel alone for 6 months should be administered in addition to oral anticoagulant.

*ACE* Angiotensin-Converting Enzyme, *BP* Blood Pressure, *CCBs* Calcium Channel Blockers, *CCD* Chronic Coronary Disease, *CVD* Cardiovascular Disease, *GDMT* Guideline-Directed Medical Therapy, *GLP-1* Glucagon Like Peptide-1, *LVEF* Left Ventricular Ejection Fraction, *MACE* Major Adverse Cardiovascular Event, *MI* Myocardial Infarction, *QOL* Quality of life, *RAS* Renin-Angiotensin System, *SGLT-2* Sodium Glucose Transporter-2, *STEMI S-T* Segment Elevation Myocardial Infarction.

#### Statins and Ezetimibe

The Treating to New Target (TNT) trial revealed that moderate (10 mg) to high intensity (80 mg) atorvastatin dosage decreases cardiovascular events when achieving LDL-C levels below 100 mg/dl in CCD patients. The cardiovascular event rate was significantly lower in individuals taking atorvastatin 80 mg compared to 10 mg, with a mean LDL-C reduction of almost 30 points^33^. Interestingly, the LODESTAR study demonstrated that adopting a high-intensity statin strategy to obtain a 50% LDL-C reduction is as effective as using an LDL-C goal of 50–70 mg/dl approach to decrease mortality, MI, stroke, and revascularization in patients with CCD. The average LDL-C level at three years was 68.4 mg/dl in the high-intensity strategy and 69.1 in the group with a target approach^34^.

Even though the LDL-C reduction is one of the objectives of statin therapies, it is essential to point out that statins may have special effects, such as endothelial function improvement, inflammation reduction, and less prothrombotic activation^35–38^. These benefits seen with statins are better known as pleiotropic effects and explain the broad utilization of these medications in cardiovascular diseases.

Ezetimibe is a synthetic drug that blocks cholesterol absorption, specifically at the small intestine’s brush border. This blocking process is facilitated by modifying the activity of the sterol transporter known as Niemann-Pick C1-Like-1 (NPC1L1), ultimately leading to its cholesterol-reducing effects^39^. The IMPROVE-IT clinical trial demonstrated that including ezetimibe alongside a moderate-intensity statin regimen resulted in a 24% reduction in LDL-C levels. Moreover, this approach contributed to a 2% absolute decrease in MACE. This trial stands as a pivotal milestone in the field of lipid management. Nonetheless, it is worth mentioning that this study did not compare this strategy with a high-intensity statin regimen^40^. According to the 2023 guidelines established by AHA/ACC, individuals with clinical atherosclerotic cardiovascular disease already taking the highest or tolerated statin dose are at a notably increased risk of cardiovascular events if their LDL-C levels are ≥70 mg/dL and they have various additional high-risk factors as discussed in the guideline document. In these cases, the implementation of ezetimibe therapy is considered beneficial (Class 2a)^2^.

The synergistic effect of statins and ezetimibe contributes to a comprehensive approach in managing lipid profiles for individuals with CCD, ultimately aiming to minimize the risk of cardiovascular events and enhance overall cardiac health.

#### PCSK-9 inhibitors

PCK-9 inhibitors, evolocumab, and alirocumab, have been linked to a potent LDL-C reduction that benefits patients with cardiovascular diseases^41,42^.

In the FOURIER trial, the primary endpoint was the combination of cardiovascular mortality, MI, stroke, hospitalization for unstable angina, or coronary revascularization. The results of this study have demonstrated that the inclusion of evolocumab in a statin treatment plan among patients with established atherosclerotic cardiovascular disease is associated with a reduction in the incidence of the primary endpoint compared to placebo (9.8% vs. 11.3%; hazard ratio, 0.85; 95% CI, 0.79 to 0.92; *P* < 0.001)^43^. However, it is worth noting that this trial had a shorter duration of follow-up as compared to other lipid-lowering trials. This may ultimately limit some of the results with regards to the long-term efficacy of evolocumab for lowering LDL-C levels.

In the PACMAN-AMI trial, utilizing alirocumab combined with rosuvastatin reduced coronary plaque thickness in patients diagnosed with acute MI after 13 months of treatment^44^. The ODYSSEY Outcomes study further supports these results, demonstrating the effectiveness of alirocumab when combined with high-intensity statins in reducing the risk of stroke, MI, cardiovascular mortality, or hospitalization for unstable angina, especially in individuals with an initial LDL-C > 100 mg/dl that achieved an LDL-C level of 25–50 mg/dl^45^. Both trials offered some insight into the efficacy of PCSK-9 inhibitors for LDL-C control when used in combination with statins in patients with CCD. However, the PACMAN-AMI trial was limited by a small sample size and both trials did not include many patients on ezetimibe therapy. Nevertheless, their results demonstrated consistent short-term efficacy of PCSK-9 inhibitors for LDL-C reduction.

While current evidence supports the efficacy of PCSK-9 inhibitors in significantly lowering LDL-C levels, their long-term impact on cardiovascular outcomes and the potential for additional benefits beyond lipid modification remain subjects for exploration. Future research may delve into the durability of the cardiovascular risk reduction observed with PCSK-9 inhibitors, their effects on atherosclerotic plaque composition, and their role in specific patient populations. Additionally, questions surrounding the cost-effectiveness and practicality of widespread PCSK-9 inhibitor use in CCD management warrant further investigation. As ongoing clinical trials and real-world data continue to emerge, the evolving understanding of PCSK9 inhibitors is likely to shape their position in the comprehensive approach to treating CCD.

#### Inclisiran

Inclisiran is a type of small interfering ribonucleic acid (RNA) that is designed to target PCSK9. By blocking the translation of the messenger RNA for PCSK9, it can effectively reduce the level of the protein and lower LDL-C concentrations^46^. Inclisiran has been tested in populations with heterozygous familial hypercholesterolemia showing prominent LDL-C reduction compared to placebo^47^. The 2023 AHA/ACC guidelines recommend that inclisiran may be an option to further lower LDL-C levels if ezetimibe and PCSK-9 inhibitors are not effective or cause adverse effects^2^. Ongoing studies, such as the ORION-4 trial, will help us elucidate the benefits of inclisiran in terms of MACE among individuals with CCD^48^.

Inclisiran offers a unique approach to sustained LDL-C reduction. Preliminary clinical trials have shown its efficacy in lowering cholesterol levels, thereby presenting potential benefits for CCD patients. The convenient dosing schedule of inclisiran, with twice-yearly injections, enhances patient adherence compared to traditional lipid-lowering therapies. The ongoing exploration of inclisiran’s long-term cardiovascular outcomes and its impact on atherosclerotic plaque stabilization provides valuable insights into its role in CCD. Future directions may involve investigating its potential synergies with existing therapies, exploring its effects in diverse patient populations, and assessing its overall impact on cardiovascular morbidity and mortality. As research unfolds, inclisiran holds promise as a valuable addition to the armamentarium for treating CCD.

#### Bempedoic Acid

Bempedoic acid is a prodrug that acts as a cholesterol-lowering treatment, primarily prescribed for individuals who exhibit intolerance to statins, or it can be used as an additional therapy alongside statins and other lipid-lowering drugs to achieve desired LDL-C levels^49,50^. This medication prevents cholesterol synthesis within the liver by inhibiting adenosine triphosphate (ATP) citrate lyase. Furthermore, it increases the expression of LDL receptors on hepatic cell surfaces, thereby facilitating LDL-C clearance from the bloodstream^51^.

In the context of the CLEAR trial, bempedoic acid exhibited notable efficacy in primary and secondary cardiovascular prevention among individuals who either experienced intolerance to statins or were reluctant to utilize them. The trial demonstrated a 13% reduction in the composite primary endpoint, including cardiovascular-related mortality, MI, stroke, and coronary revascularization events. Moreover, using bempedoic acid resulted in favorable outcomes, showing a lowered incidence of cardiovascular mortality, stroke, and MI compared to the placebo group. Additionally, bempedoic acid demonstrated marked 23% and 19% reductions in fatal and nonfatal MI and coronary revascularization events, respectively^52^.

Even though no trial comparing the efficacy of bempedoic acid and PCSK-9 inhibitors exists, in a phase II RCT, the combination of these therapies improved the lipid profile among patients with hyperlipidemia, notably decreasing LDL-C levels in comparison with placebo^53^. Further studies are needed to understand if PCSK-9 inhibitors can be used as the primary alternative in the case of statin intolerance, given the high cost of bempedoic acid. There are also concerns related to bempedoic acid’s long-term efficacy, potential side effects, and questions regarding its comparative efficacy with established therapies.

#### Icosapent ethyl

Icosapent ethyl is a stable and extensively refined eicosapentaenoic acid (EPA) form. It has a range of beneficial impacts, including reducing inflammation, mitigating oxidative stress, and stabilizing plaques and cell membranes. This therapy is indicated for patients with hypertriglyceridemia with or without elevated LDL-C^54–57^. The Japan EPA Lipid Intervention Study (JELIS) was a clinical trial that explored the impact of FAs. The study showed that using EPA in patients with CCD and hypercholesterolemia led to a significant decrease in major coronary events, but this effect was not present in patients without a history of CCD^58^.

The STRENGTH study revealed that supplementing carboxylic acid formulation of EPA/DHA (Omega-3 CA) to individuals with high cardiovascular risk already on statin treatment did not reduce combined MACEs^59^. In addition, the 2023 AHA/ACC Guidelines do not recommend carboxylic acid Omega-3 supplements (Class III)^2^. In the Study of AMR101 to Evaluate Its Ability to Reduce Cardiovascular Events in High-Risk Patients With Hypertriglyceridemia and on Statin (REDUCE-IT) clinical trial, the administration of icosapent ethyl at a dosage of 2 grams twice a day in conjunction with moderate-high intensity statins resulted in a noteworthy 25% reduction in the likelihood of experiencing the primary outcome composed by MI, nonfatal stroke, coronary revascularization, and stable angina, in comparison to the placebo that contained mineral oil. It is important to highlight that more than two-thirds of the participants in the trial had a confirmed history of cardiovascular disease. Moreover, this trial was notably limited by the fact that very few patients included were on ezetimibe or PCSK-9 inhibitor therapy. Regardless, icosapent ethyl demonstrated a marked 26% in the study’s secondary outcome, including cardiovascular mortality, MI, or stroke^60^. The Effect of Vascepa on Improving Coronary Atherosclerosis in People With High Triglycerides Taking Statin Therapy (EVAPORATE) trial established that patients with hypertriglyceridemia and confirmed coronary stenosis who received icosapent ethyl, in addition to statin therapy, experienced a significant reduction in the volume of low-attenuation plaque, which helps stabilize the plaque and prevent its rupture^61^. However, one must recognize that this trial was limited by a small sample size and inadequate power to assess for long-term outcomes.

For individuals with CCD who are already on maximum tolerated statin therapy and have an LDL-C level < 100 mg/dl, it is recommended by the AHA/ACC Guidelines to add icosapent ethyl if they have persistent fasting triglyceride levels between 150–499 mg/dl after ruling out any secondary causes that can explain this elevation. This strategy can help reduce the risk of MACE and cardiovascular mortality^2^.

While there appears to be some cardiovascular risk reduction when icosapent ethyl is added to statin therapy, there are remaining questions regarding its optimal placement in treatment algorithms, cost implications, and potential side effects. Additionally, it is unclear whether it has any role as monotherapy. Future directions may involve further research to delineate the specific patient populations that would benefit the most from icosapent ethyl, exploring its long-term safety profile, and evaluating its role as monotherapy or in combination with other emerging therapies for CCD. Clarifying these aspects will contribute to a more nuanced understanding of icosapent ethyl’s place in the evolving landscape of CCD management.

#### Other supplements (Omega 3, fish oil, and vitamins)

Omega-3 fatty acids have emerged as a subject of considerable interest in cardiovascular health, especially concerning CCD. Extensive data has revealed a spectrum of potential advantages linked to the intake of FAs, placing them as a promising therapy for managing this condition. A meta-analysis showed that omega-3 fatty acids demonstrated that FAs are linked to decreased cardiovascular mortality and other related outcomes. The reduction in cardiovascular risk was more significant when using EPA alone compared to a combination of EPA and docosahexaenoic acid (DHA)^62^. However, the risk of atrial fibrillation is more significant in those taking FAs^63^.

There is no evidence to suggest that vitamins are effective in treating CCD. A meta-analysis revealed that vitamin D supplements do not decrease MACE and other cardiovascular outcomes like MI, stroke, cardiovascular disease-related death, or overall mortality. As a result, vitamin D supplementation does not provide any benefits for cardiovascular protection and should not be recommended for such a purpose^64^. Some meta-analyses have indicated that Vitamin C, whether used alone or combined with Vitamin E and beta-carotene, did not impact CCD or other significant cardiovascular events^65,66^.

The ACC/AHA Guidelines recommend that people with CCD avoid over-the-counter or dietary supplements like fish oil, omega-3 fatty acids, vitamins, and calcium supplements. This advice is based on the absence of confirmed advantages in lowering cardiovascular events among patients with CCD^2^.

While some of these dietary supplements may be considered for their potential benefits in CCD management, several concerns merit careful consideration. One primary concern revolves around the lack of standardized regulation for supplements, leading to variations in product quality and potency. Inconsistent formulations and potential contaminants may compromise the efficacy and safety of these supplements. Moreover, relying on supplements may divert attention from the broader lifestyle modifications recommended for CCD, such as a heart-healthy diet, regular exercise, and medication adherence. Excessive intake of certain supplements, particularly antioxidants and vitamin supplements, may pose risks, with studies suggesting potential adverse effects on cardiovascular health. Additionally, interactions between supplements and prescribed medications could lead to unintended consequences. It is crucial to approach supplement use judiciously, with a focus on evidence-based recommendations, close monitoring for potential side effects, and collaboration with healthcare professionals to ensure a comprehensive and safe approach to CCD management.

### Beta blockers

Current evidence strongly supports the use of beta blockers in managing CCD^1,2^. These medications effectively reduce cardiac workload by slowing heart rate and lowering blood pressure. Beta-blockers dilate coronary arteries, improve oxygen supply to cardiac muscle, and lower oxygen demand^67^. When selecting a beta blocker for a patient with CCD, it is essential to carefully consider factors such as the intrinsic medication properties, the appropriate dosage, and the characteristics of the individual to maximize therapeutic benefits while minimizing potential side effects^2^.

According to the ACC/AHA Guidelines, long-term beta-blocker use is not currently recommended to improve outcomes in people with CCD unless there is a documented history of MI in the past year, the ejection fraction of the left ventricle is 50% or lower, or there is another specific reason to use beta-blockers (e.g. tachycardia). In the context of acute-onset angina management, it is advisable to consider the potential benefits of incorporating a calcium channel or beta blocker into the treatment regimen of CCD^2^ (Table 1).

Overall, beta blockers remain one of the mainstays of CCD treatment given their various benefits such as cardioprotection, antiarrhythmic effects, and secondary prevention. They reduce heart rate, myocardial oxygen demand, and blood pressure, preventing angina and myocardial infarctions. However, it is important to recognize some of their potential side effects as well. These include the risk for potential bronchoconstriction, masking of hypoglycemic symptoms, and the risk of sexual dysfunction, fatigue, and exercise intolerance. While these effects are usually well tolerated, the decision to prescribe beta-blockers should be personalized, considering individual patient characteristics, comorbidities, and potential side effects, with ongoing monitoring to optimize benefits and minimize drawbacks.

### Antiplatelet therapy

There is ongoing debate regarding the optimal length of antiplatelet therapy for patients with CCD. Clinicians have the option to choose between a monotherapy strategy that employs a single antiplatelet therapy (SAPT), such as aspirin or clopidogrel, or a dual antiplatelet therapy (DAPT) that usually involves combining aspirin with a P2Y12 inhibitor. The goal is to balance the benefit of reducing the risk of ischemic events and the development of bleeding in individuals with CCD^68^. According to the CHARISMA trial, patients with symptomatic atherothrombosis may benefit from clopidogrel treatment. However, adjunctive clopidogrel use was associated with significantly higher rates of moderate bleeding and a signal towards severe bleeding, albeit statistically insignificant, in patients with established cardiovascular disease. A statistically insignificant trend towards moderate and severe bleeding was also seen with clopidogrel use in patients with a history of multiple atherothrombotic risk factors. Furthermore, the combined use of clopidogrel and aspirin did not show significant improvement in reducing the occurrences of MI, stroke, or cardiovascular-related mortality in comparison to aspirin usage alone^69^.

Another important consideration in antiplatelet therapy is the lack of clarity regarding the transition from ACS to CCD. The transition point from ACS to CCD is not concretely defined by a specific time frame and there is a paucity of evidence concerning the appropriate distinction. Regardless, most consider 1 year to be the transition point. And while the ACC/AHA Guidelines do not make a specific distinction, they do recognize that care for CCD is a continuum from post-acute care of patients with ACS to outpatient CCD management. It is also important to recognize that the optimal duration and intensity of antiplatelet therapy become complex as patients may move between these phases. Balancing the need for potent antiplatelet agents in the acute setting with the potential risks of long-term therapy in stable coronary disease poses a clinical dilemma. The risk of bleeding becomes particularly relevant in the chronic phase when the benefits of continued therapy may be less pronounced.

Several RCTs and meta-analyses suggest that short-term DAPT duration (generally between 3–6 months) following SAPT is the best approach to treat patients with CCD^70–75^. The extended duration of DAPT is typically characterized as a period exceeding 12 months^68^. Although one meta-analysis and a randomized trial (PEGASUS) showed that prolonged DAPT decreases the risk of MI, this approach was associated with an increased incidence of bleeding events^76,77^.

Based on the ACC/AHA Guidelines^2^, adopting shorter durations of DAPT can be considered a viable and effective approach among diverse scenarios, mainly when there is a significant risk of bleeding and a low to moderate probability of experiencing ischemic events^2^. In fact, the guidelines recommend that CCD patients treated with percutaneous coronary intervention (PCI) complete 6 months of DAPT (aspirin and clopidogrel) followed by SAPT to reduce MACE and bleeding events (Class I, LOE A). At the same time, the guidelines do give a class 2a (LOE A) recommendation for these same patients to be treated with DAPT for 1 to 3 months, followed by P2Y12 monotherapy for at least 12 months. Patients with CCD and no indication for oral anticoagulation are also recommended to take low-dose aspirin to reduce atherosclerotic events (Class 1, LOE A). For CCD patients who do require anticoagulation and have undergone PCI, the recommendation is for triple therapy for 1 to 4 weeks, followed by clopidogrel and anticoagulation therapy for 6 months.

The 2019 ESC guidelines^1^ on CCD similarly recommend daily low-dose aspirin for patients with prior MI or revascularization (Class I; LOE A), but they do notably add clopidogrel as an acceptable alternative (Class I; LOE B). Like the ACC/AHA guidelines, the ESC guidelines recommend DAPT (aspirin and clopidogrel) for 6 months following PCI in CCD. The ESC guidelines also give a IIa recommendation for 3 months of aspirin plus clopidogrel after PCI if there is concern for a high bleeding risk profile, but they do give a class IIb recommendation for 1 month of DAPT following PCI (Class IIb, LOE C). For CCD patients with an indication for anticoagulation, the ESC guidelines recommend the continued use of aspirin, clopidogrel plus oral anticoagulant with early cessation of aspirin at ≤ 1 week (Class IIa; LOE B). A more delayed discontinuation of aspirin (≥1 month) can be considered as well in circumstances where the risk of thrombosis outweighs the risk of bleeding (Class IIa; LOE C).

Several unanswered questions remain in the realm of antiplatelet therapy for CCD. While these medications, particularly aspirin and P2Y12 inhibitors, play a pivotal role in preventing cardiovascular events, the optimal duration and combinations of such agents remain uncertain. The balance between the benefits of reducing thrombotic events and the potential for bleeding complications poses a critical query, especially in long-term usage. Additionally, the question of triple therapy, combining antiplatelet agents with anticoagulation in patients with CCD and a condition warranting systemic anticoagulation, raises concerns about bleeding risks and the optimal duration of such regimens. The delicate equilibrium between preventing ischemic events and avoiding hemorrhagic complications adds complexity to the management strategy. Moreover, the role of newer antiplatelet agents and their efficacy compared to traditional therapies remains an area of exploration. Understanding the nuances of patient selection, duration of treatment, and the interplay between antiplatelet agents and evolving anticoagulant strategies represents a focal point for ongoing research.

### Glucagon-like peptide-1 (GLP-1) receptor agonists and sodium-glucose transporter 2 (SGLT-2) inhibitors

The utilization of GLP-1 receptor agonists and SGLT-2 inhibitors is crucial for effectively managing CCD. These relatively novel therapeutic agents exert their effects by primarily targeting the reduction of glucose levels while positively influencing cardiovascular health across several mechanisms. Specifically, GLP-1 receptor agonists have been demonstrated to enhance LV function^78^, possibly enabling coronary vasodilation^79^, reducing oxidative stress^80^, and stabilizing arterial plaques^81^. These agents also enhance cardiovascular outcomes for diabetic patients by decreasing the incidence of MI and cardiovascular-related deaths^82–84^. The mechanism behind their efficacy is multifold. There is a growing body of evidence indicating that GLP-1 receptors are also expressed in cardiomyocytes and vascular endothelial cells^85^. The GLP-1 receptor agonists, therefore, can exhibit cardioprotective effects by promoting myocardial glucose uptake, reducing oxidative stress, and inhibiting cardiomyocyte apoptosis, ultimately preventing adverse cardiac remodeling. In addition, the activation of GLP-1 receptors induces vasodilation through the stimulation of endothelial nitric oxide production, improving coronary blood flow, and potentially modulating the renin-angiotensin-aldosterone system^85^. Additionally, GLP-1 agonists consistently lead to weight loss through various mechanisms, contributing to improved insulin sensitivity and reduced overall cardiovascular risk. Ultimately these agents exert anti-inflammatory and antiatherogenic effects, reducing the formation and progression of atherosclerotic lesions^86^. Beyond glycemic control, GLP-1 receptor agonists demonstrate a multifaceted impact on the cardiovascular system, encompassing direct cardioprotection, vasodilation, natriuresis, weight reduction, lipid profile improvement, anti-inflammatory effects, renal protection, and neurohormonal regulation. These findings underscore the diverse potential of GLP-1 receptor agonists in reducing cardiovascular risk and hold significant implications for the tailored treatment of patients with CCD.

Many RCTs and systematic meta-analyses have found evidence of significant cardiovascular risk reduction and safety with the use of GLP-1 agonists in patients with diabetes and a history of cardiovascular disease. More specifically, this risk reduction appears to be primarily a reduction in the risk of atherosclerotic events, such as MI and stroke. In the Liraglutide Effect and Action in Diabetes: Evaluation of Cardiovascular Outcome Results (LEADER) trial, there was a significantly lower rate of first occurrence of death from cardiovascular causes, nonfatal MI, or nonfatal stroke among patients with type 2 diabetes mellitus with GLP-1 agonist than with placebo^87^. Similar results were noted in the Trial to Evaluate Cardiovascular and Other Long-term Outcomes With Semaglutide in Subjects With Type 2 Diabetes (SUSTAIN-6) and Researching Cardiovascular Events With a Weekly Incretin in Diabetes (REWIND) trials^88,89^. While the Evaluation of Cardiovascular Outcomes in Patients With Type 2 Diabetes After Acute Coronary Syndrome During Treatment With AVE0010 (ELIXA) and Exenatide Study of Cardiovascular Event Lowering Trial (EXSCEL) trials did not find significant MACE reduction with GLP-1 receptor agonists, they did note cardiovascular safety of GLP-1 agonist use within this high-risk population^90,91^. There are several other trials in this high-risk population of diabetic patients that have demonstrated cardiovascular benefits with GLP-1 use^92–94^. While new evidence is emerging for GLP-1 agonists, there is much to be learned regarding their use in combination with other cardiovascular or diabetes medications to enhance cardioprotective effects. Additionally, there is a need for extended studies to assess the long-term safety, cost-effectiveness, and tolerability of GLP-1 agonists. Furthermore, there is an interest in expanding research to non-diabetic populations with cardiovascular risk factors to explore the potential benefits of GLP-1 agonists beyond its anti-diabetic properties.

In contrast to GLP-1 agonists, SGLT-2 inhibitors appear to exert their beneficial cardiovascular effects via a reduction in incident and worsening heart failure and slowing of kidney disease decline in individuals with CCD^95^. The beneficial effects of SGLT2 inhibitors can be associated with a group of mechanisms, including early natriuresis, enhanced vascular function, and reduced blood pressure. Additionally, these medications may help alleviate inflammation caused by fatty tissue, lowering pro-inflammatory cytokines. They can also shift metabolism towards ketone bodies, decreasing oxidative stress, serum uric acid, glomerular hyperfiltration, and albuminuria^96^. Several major trials, including Empagliflozin, Cardiovascular Outcomes, and Mortality in Type 2 Diabetes (EMPA-REG OUTCOME), Canagliflozin Cardiovascular Assessment Study (CANVAS Program), Dapagliflozin Effect on Cardiovascular Events (DECLARE-TIMI 58), Canagliflozin and Renal Events in Diabetes with Established Nephropathy Clinical Evaluation (CREDENCE), and Effect of Ertugliflozin on Cardiovascular Outcomes in Type 2 Diabetes Mellitus Participants With Vascular Disease (VERTIS CV), have investigated the cardiovascular benefits of SGLT-2 inhibitors in patients with type 2 diabetes^97–101^. These trials consistently demonstrated significant cardiovascular risk reduction benefits. These included reductions in MACEs, cardiovascular death, heart failure hospitalizations, and even MI and stroke. Collectively, these trials provide compelling evidence supporting the cardiovascular benefits of SGLT2 inhibitors in patients with type 2 diabetes. However, these studies were not specifically focused on patients with baseline CCD. More recently, a systematic meta-analysis compared SGLT-2 inhibitor treatment with placebo or other glucose-lowering treatment in comprehensive outcomes of cardiovascular events and cardiorenal parameters in patients with known CCD. The analysis ultimately demonstrated that in patients with CCD, SGLT-2 inhibitors significantly reduced cardiovascular events compared with the placebo^102^. Nevertheless, further validation of SGLT-2 inhibitor use in the setting of CCD is needed in future RCT studies.

Presently, there is insufficient compelling evidence to suggest that either medication reduces cardiovascular risk in patients with combined cardiovascular disease but without type 2 diabetes. Regardless, according to the 2023 ACC/AHA Guidelines, it is advised to consider the utilization of SGLT-2 inhibitors and GLP-1 receptor agonists for specific groups of individuals with CCD (Table 1). This recommendation applies even to those patients without diabetes^2^. GLP-1 receptor agonists and SGLT-2 inhibitors offer a range of benefits, making them excellent therapies to be considered for optimizing the management of CCD. Despite the effectiveness of GLP-1 agonists and SGLT-2 inhibitors in CCD, their integration into clinical practice has been slow, emphasizing the potential for cardiovascular specialists to play a more significant collaborative role in the management of patients with combined CCD and type 2 diabetes.

### Antianginal therapy

Antianginal therapies relieve angina episodes among patients with CCD by decreasing the oxygen demand (beta-blockers [mentioned above], calcium channel blockers [CCBs]) or increasing the amount of oxygen to the cardiac muscle (nitrates and dihydropyridine CCBs)^103^. Ranolazine, another antianginal medication, may inhibit the late sodium current and reduce calcium overload, but its action mechanism still needs to be fully understood^104^. The primary objective of treatment is to alleviate symptoms without exacerbating comorbidities, reducing potential interactions with other medications, and guaranteeing the patient’s ability to tolerate the treatment^2^.

In the case of antianginal therapy for CCD, treatment success is measured by the relief of symptoms. In some cases, one medication may be more appropriate than another, such as using a beta blocker for a patient with concomitant left ventricular dysfunction. Most patients can achieve symptom control with these treatments. However, relief from angina is only possible for 40% to 50% of patients, depending on the frequency of angina at the beginning of treatment^105^.

### Renin Angiotensin Aldosterone inhibitors

According to the ACC/AHA guidelines, renin angiotensin aldosterone (RAAS) inhibitors, such as Angiotensin-converting enzyme (ACE) inhibitors and Angiotensin receptor blockers (ARBs), are indicated in individuals with CCD in the context of hypertension, diabetes, chronic kidney disease or LV ejection fraction ≤ 40% (Class I)^2^. This recommendation is largely in line with the ESC guidelines which advise the use of ACE inhibitors or ARBs for CCD patients with concomitant heart failure, hypertension, or diabetes (Class I, LOE A)^1^. They also recommend consideration of ACE inhibitors and ARBs in CCS patients at very high risk of cardiovascular events (Class IIa; LOE A). RAAS inhibitors have demonstrated several benefits in these patient populations through their pleiotropic mechanisms of vasodilation, inhibition of sodium and water retention, and attenuation of cardiac remodeling^106,107^. In fact, several RCTs have demonstrated improvements in symptoms, reduced hospitalizations, and prolonged survival among high-risk patients with CCD^108–112^. Many of the patients across these trials had evidence of left ventricular dysfunction with a history of prior MI. The Heart Outcomes Prevention Evaluation (HOPE) trial also evaluated high-risk CCD patients (age > 55 years) with existing or previous cardiovascular disease, or diabetes. The trial found a significant benefit from ramipril treatment in reducing the combined primary endpoint of cardiovascular death, non-fatal MI, and non-fatal stroke^113^.

However, it is worth noting that RCTs in lower-risk CCD patients have had inconsistent results. For instance, the EURopean trial On the reduction of cardiac events with Perindopril in stable coronary Artery disease (EUROPA) trial found no mortality benefit when using an ACE inhibitor in patients with CCD and normal systolic function^114^. Additional studies like the Quinapril Ischemic Event trial (QUIET) and the Comparison of Amlodipine versus Enalapril to Limit Occurrences of Thrombosis (CAMELOT) trial found no significant benefits in MACE reduction with RAAS inhibiton^115,116^. A large meta-analysis by Bangalore et al., including 24 trials and over 60,000 patients, found that RAAS inhibitors in CCD patients without heart failure only reduced cardiovascular events and death when compared to placebo, but not when compared to active controls^117^. As such, both guidelines do not give strong recommendations for RAAS inhibitors in CCD patients without heart failure or other major cardiovascular risk factors.

The lack of overall benefit in these lower-risk populations could be explained by several factors. Firstly, lower-risk populations may have fewer cardiovascular events overall, making it challenging to demonstrate a statistically significant reduction in MACE with RAAS inhibition. Secondly, because low-risk patients have fewer baseline comorbidities, the potential benefit of RAAS inhibition may be less pronounced in the absence of additional risk factors. Moreover, low-risk patients may already be receiving other evidence-based therapies, such as statins, antiplatelet agents, and beta-blockers, which could contribute to optimal cardiovascular risk reduction. The additional benefit of RAAS inhibitors in this context may be limited. There is also the possibility that the underlying disease progression and pathophysiology in low-risk CCD might differ from that in high-risk patients. RAAS inhibition therefore can have a more substantial impact in populations with advanced disease or higher cardiovascular risk. Lastly, CCD is a heterogeneous condition, and the response to RAAS inhibitors may vary based on specific subtypes of CCD.

## Revascularization

Revascularization procedures, encompassing both PCI and coronary artery bypass grafting (CABG), represent important therapeutic strategies in the management of CCD because they can significantly reduce symptoms, prevent cardiac events, and possibly improve mortality in select patients. However, while both the ESC and ACC/AHA guidelines recognize revascularization as an important therapy for CCD patients, they view it as an adjunct treatment to optimal medical therapy^1,2^.

It has been well established that revascularization when compared to medical therapy, results in significant improvement in quality of life and angina. This was evident in the 5-year follow-up of the FAME 2 (Fractional Flow Reserve versus Angiography for Multivessel Evaluation 2) trial, wherein revascularization improved quality of life, and reduced the use of antianginal drugs and associated side effects for patients with CCD^118^. These improvements in symptoms and quality of life with revascularization (both with PCI or CABG) in CCD were further supported by the results from the ISCHEMIA (International Study of Comparative Health Effectiveness With Medical and Invasive Approaches), EXCEL (Evaluation of XIENCE Versus Coronary Artery Bypass Surgery for Effectiveness of Left Main Revascularization), BARI2D (Bypass Angioplasty Revascularization Investigation in Type 2 Diabetes) and COURAGE (Clinical Outcomes Utilizing Revascularization and Aggressive Drug Evaluation) trials^119–122^. However, it is worth mentioning the shared limitations of these trials. For instance, the follow-up durations for these trials were relatively short, ranging from a few years to approximately five years, possibly missing longer-term insights and the durability of interventions like CABG. Additionally, the trials were conducted during periods of evolving medical therapies, and the definition of optimal medical therapy may not fully align with contemporary standards. Additionally, technological advancements and changes in interventional cardiology practices may have occurred since the trials were conducted. Moreover, the trial populations were primarily from North America and Europe, raising concerns about generalizability to other geographic regions. Notable crossover rates between treatment groups during follow-up, limitations in assessing quality of life and symptoms, and potential impacts of changes in clinical practice patterns over time are additional considerations when interpreting their results. Most recently, however, the results from the Placebo-controlled Trial of Percutaneous Coronary Intervention for the Relief of Stable Angina (ORBITA-2) trial also found that PCI in CCD patients with stable angina (on little or no antianginal therapy) resulted in significantly less anginal symptoms when compared to placebo^123^. This was the first placebo-controlled trial to investigate this question. While this trial was limited by a short duration of follow-up and a relatively small sample size, it did provide strong evidence in support of the symptomatic benefits of PCI for patients with stable ischemic heart disease.

Revascularization, when pursued in appropriate patients with CCD, can also potentially lower the risk for cardiovascular death, MI and urgent revascularization, especially amongst patients with higher-risk CAD. For example, in MASS II (Medicine, Angioplasty, or Surgery Study), the 10-year rates of cardiac death were lower after CABG or PCI than after medical therapy alone^124^. Similarly, the ISCHEMIA trial demonstrated a reduction in the rates of cardiovascular mortality with revascularization in CCD patients at 7-year follow-up^121^. The trial also found a reduced incidence of spontaneous type 1 and type 2 MIs in the revascularized CCD patients. More recent meta-analyses have also demonstrated a reduction in spontaneous MI with revascularization in CCD patients.

While revascularization offers benefits with regard to symptom improvement and cardiovascular event reduction, there is no consistent all-cause mortality benefit noted in RCTs and meta-analyses. Several meta-analyses and studies have noted an overall null effect of revascularization on all-cause death in CCD patients^125–128^. However, it is worth noting that more contemporary trials and studies are warranted, given the significant number of high crossover rates and low angiographic complexity patients in these trials. Additionally, much of the data comes from older studies, and the currently available revascularization techniques for CABG and PCI have seen numerous technical advancements. Despite the evidence showing no significant mortality benefit with revascularization in the general CCD population, there is some data to support a mortality benefit for CABG amongst specific CCD subgroups. For example, CABG demonstrates a mortality benefit over medical therapy alone in two specific subgroups: patients with multivessel CAD and moderate to severe left ventricular dysfunction (ejection fraction ≤35%), as well as those with left main coronary disease. In CASS (Collaborative Study in Coronary Artery Surgery), patients with left main coronary artery disease had significantly higher three-year survival rates with CABG compared to medical treatment. The difference was significant across various levels of left ventricular function and for patients with stenosis of the left main coronary artery^129^. Similarly, the STICH (Surgical Treatment for Ischemic Heart Failure) trial revealed that individuals with ischemic cardiomyopathy and reduced LV ejection fraction (≤35%) who underwent CABG in conjunction with optimal medical therapy experienced enhanced survival over a 10-year period compared to those receiving medical therapy alone^130^. In contrast, the REVIVED-BCIS2 trial found that PCI in CCD patients with ejection fraction ≤35%, did not yield improvement in survival when compared to optimal medical therapy alone^131^.

Given the aforementioned evidence, the ACC/AHA guidelines recommend pursuing revascularization to improve symptoms in patients with CCD and lifestyle-limiting angina despite guideline-directed medical therapy and with significant CAD that is amenable to revascularization (Class I; LOE A)^2^. They also recommend CABG in CCD patients with significant left main or multivessel disease with an EF < 35% to improve survival. No other class I indications for revascularization are provided in the guidelines.

Overall, revascularization strategies in CCD present a complex set of issues that necessitates careful consideration. While interventions such as PCI or CABG aim to alleviate ischemic symptoms and improve outcomes, challenges persist. The appropriateness of revascularization procedures requires meticulous assessment of individual patient characteristics, lesion complexity, and the extent of coronary artery involvement. Deciding between PCI and CABG involves weighing factors like patient comorbidities, lesion location, and anatomical considerations. In CCD, where atherosclerosis is often diffuse and involves multiple vessels, determining the optimal revascularization strategy becomes particularly challenging. Issues related to long-term efficacy, the potential for restenosis, and the need for repeat procedures underscore the complexity of decision-making in revascularization for CCD. Striking a balance between the invasiveness of the procedure and its potential benefits remains a critical consideration, necessitating a personalized approach informed by evolving research and clinical guidelines.

## Future directions

There is significant potential for future directions in the diagnosis and management of CCD. Ultimately, the success of novel approaches hinges on targeted advancements in both pharmacological and nonpharmacological strategies. The future of managing CCD requires a comprehensive research agenda, addressing evolving challenges and opportunities across the spectrum of diagnosis, risk stratification, and treatment. For instance, noninvasive and invasive imaging modalities enabling precise detection and quantification of atherosclerotic plaque burden and assessment of plaque characteristics (degree of calcified vs. noncalcified), warrant further investigation for their impact on patient identification, prognosis, and eligibility for preventive therapies. These include modalities such as cardiac computed tomography angiography (CCTA), intravascular ultrasound (IVUS), optimal coherence tomography (OCT), and positron emission tomography (PET). Each of these provides its own unique advantages and can significantly aid in the identification of high-risk CCD lesions. Similarly, the development and validation of comprehensive risk scores for MACEs in CCD patients, incorporating demographics, medical comorbidities, social determinants, and noninvasive or invasive test results, are crucial for contemporary risk assessment. A study by Blaum et al. in 2021 investigated the predictive ability of various risk scores for predicting MACEs in CCD patients and noted only modest efficacy for contemporary risk scores^132^. Risk stratification may also be further bolstered by utilizing precision medicine and expanding insights into the roles of genomics and proteomics for disease pathology. Regarding therapies, there is a significant need to explore the utility of GLP-1 for the treatment of CCD in patients without diabetes. While there is some data suggesting a reduction in MACEs for non-diabetic patients on GLP-1 agonists, randomized clinical trial data is lacking^133^. Additionally, optimal duration and regimen selection of antiplatelet and antithrombotic agents for patients with CCD are continually being refined to better balance the cardiovascular benefits with the risks of major bleeding events. Lastly, there is significant innovation underway in the field of interventional cardiology with regards to revascularization techniques and technology. The emergence of bioresorbable vascular scaffolds may represent a revolutionary device for percutaneous coronary interventions. Additionally, the advent of ultrathin drug-eluting stents offers increased flexibility and drug deliverability. There is also interest in the use of drug-coated balloons for revascularization in select patients. All in all, the future of CCD diagnosis and management remains very promising.

## Conclusions

In conclusion, CCD demands a comprehensive and multifaceted approach for prevention and management, integrating both pharmacological and nonpharmacological interventions to optimize patient outcomes. Medications, such as beta-blockers, antiplatelet agents, and novel therapies like GLP-1 agonists and SGLT-2 inhibitors, play pivotal roles in managing cardiovascular risk factors and preventing adverse events. Concurrently, lifestyle modifications, including regular physical activity, heart-healthy diets, and smoking cessation, contribute significantly to overall CCD management. However, the landscape of CCD management continues to evolve, necessitating ongoing research to address unanswered questions regarding medication regimens, antiplatelet therapy duration, and the integration of emerging therapies. Revascularization strategies, despite their challenges, remain crucial in selected cases. As we navigate the complex interplay of pharmacological and nonpharmacological interventions, future investigations must delve into refining treatment algorithms, elucidating the long-term effects of newer therapies, and tailoring approaches to individual patient profiles. The pursuit of deeper insights into these aspects will undoubtedly shape the landscape of CCD management, optimizing care and enhancing the quality of life for affected individuals.
